# Laser-Ablative Synthesis of Ultrapure Magneto-Plasmonic Core-Satellite Nanocomposites for Biomedical Applications

**DOI:** 10.3390/nano12040649

**Published:** 2022-02-15

**Authors:** Anton A. Popov, Zaneta Swiatkowska-Warkocka, Marta Marszalek, Gleb Tselikov, Ivan V. Zelepukin, Ahmed Al-Kattan, Sergey M. Deyev, Sergey M. Klimentov, Tatiana E. Itina, Andrei V. Kabashin

**Affiliations:** 1 Institute of Engineering Physics for Biomedicine (Phys-Bio), Moscow Engineering Physics Institute, 115409 Moscow, Russia; ivan.zelepukin@gmail.com (I.V.Z.); deyev@ibch.ru (S.M.D.); smklimentov@mephi.ru (S.M.K.); 2 Laboratory of Lasers Plasmas and Photonic Processing, CNRS, Aix-Marseille University (Campus of Luminy), 13288 Marseille, France; celikov@physics.msu.ru (G.T.); ahmed.al-kattan@univ-amu.fr (A.A.-K.); 3 Institute of Nuclear Physics, Polish Academy of Sciences, 31342 Kraków, Poland; zaneta.swiatkowska@ifj.edu.pl (Z.S.-W.); marta.marszalek@ifj.edu.pl (M.M.); 4 Center for Photonics and 2D Materials, Moscow Institute of Physics and Technology, 141700 Dolgoprudny, Russia; 5 Shemyakin–Ovchinnikov Institute of Bioorganic Chemistry, Russian Academy of Sciences, 117997 Moscow, Russia; 6 Hubert Curien Laboratory (UMR CNRS 5516), Jean Monnet University, 42000 Saint-Etienne, France; tatiana.itina@univ-st-etienne.fr

**Keywords:** nanoparticles, laser ablation in liquids, gold, iron, core-satellite, IR hyperthermia, modeling

## Abstract

The combination of magnetic and plasmonic properties at the nanoscale promises the development of novel synergetic image-guided therapy strategies for the treatment of cancer and other diseases, but the fabrication of non-contaminated magneto-plasmonic nanocomposites suitable for biological applications is difficult within traditional chemical methods. Here, we describe a methodology based on laser ablation from Fe target in the presence of preliminarily ablated water-dispersed Au nanoparticles (NPs) to synthesize ultrapure bare (ligand-free) core-satellite nanostructures, consisting of large (several tens of nm) Fe-based core decorated by small (mean size 7.5 nm) Au NPs. The presence of the Fe-based core conditions a relatively strong magnetic response of the nanostructures (magnetization of >12.6 emu/g), while the Au NPs-based satellite shell provides a broad extinction peak centered at 550 nm with a long tale in the near-infrared to overlap with the region of relative tissue transparency (650–950 nm). We also discuss possible mechanisms responsible for the formation of the magnetic-plasmonic nanocomposites. We finally demonstrate a protocol to enhance colloidal stability of the core-satellites in biological environment by their coating with different polymers. Exempt of toxic impurities and combining strong magnetic and plasmonic responses, the formed core-satellite nanocomposites can be used in biomedical applications, including photo- and magneto-induced therapies, magnetic resonance imaging or photoacoustic imaging.

## 1. Introduction

Recent advances in the development of nanotechnologies have led to the emergence of novel diagnostic and therapeutic modalities based on new nanomaterials [1,2,3]. Much attention in this field is given to plasmonic nanoparticles (NPs), which are capable of providing a variety of phenomena, including resonant optical extinction (absorption + scattering) in the visible and near infrared (near-IR) range [4], strong local field enhancement [5], resistance to photobleaching, and highly efficient photothermal conversion [6]. Gold (Au) NPs presents the most explored plasmonic nanomaterial [7] due to its chemical stability, biocompatibility [8], and the availability of simple surface functionalization protocols via thiol chemistry [9]. Au NPs were successfully used in therapeutic modalities, including photo-induced hyperthermia [10] and in diagnostic modalities, including optical coherence tomography [11], photoacoustic tomography [12], and confocal reflectance microscopy [13]. Magnetic NPs represent another appealing class of nanomaterials for biomedical applications [14]. These NPs can be guided by external magnetic fields both in vitro and in vivo to enable a series of therapeutic and diagnostic modalities, including magnetic resonance imaging [15], magnetic separation of cells and proteins [16], controllable drug delivery [17], and magnetic hyperthermia [18]. Among magnetic materials, iron (Fe)-based NPs are considered to be the most promising due to their biocompatibility, biodegradability, and strong magnetic response [14].

The combination of plasmonic and magnetic functionalities within a single nanoformulation looks especially interesting as it promises a synergetic enhancement of involved therapeutic and diagnostic modalities. Fe-Au magneto-plasmonic nanostructures are typically composed of a Fe-based core, covered by an Au shell (Fe@Au core-shells), which makes possible both magnetic response and the generation of plasmonic extinction peak in the visible and near-IR range [19,20]. Core-satellite structures present one of the promising variations of Fe@Au core-shell geometry, in which relatively large (>50 nm) Fe-based NPs are decorated by a layer (layers) of small (<10 nm) Au NPs [21,22,23,24,25,26] (Fe@Au core-satellites). These structures not only enable magneto-plasmonic response, but also offer a large surface for the conjugation with various functional biomolecules, as the surface area of satellite material (Au) is much larger compared to alternative structures of similar size (spherical nanoparticles, classical core-shells with completely covered core surface). However, advantages provided by the core-satellite structures are usually compromised by a high cost and complexity of synthesis procedures [21,22,23]. In fact, conventional chemical routes imply stepwise processes, in which preliminarily prepared Au and Fe oxide NPs are first functionalized by appropriate ligands, which renders possible a subsequent assembling of the core-satellites [24,25,26]. Such techniques provide relatively good control of size, composition, and structure of the Fe@Au core-satellites, but the use of ligands blocks the surfaces of Fe or Au (or both), which complicates their further conjugation with functional biomolecules and protective biopolymers. Moreover, biomedical applications of chemically synthesized NPs often have limitations due to the presence of toxic impurities originated from precursors or incomplete reactions. Another synthesis pathway is based on X-ray radiolysis of HAuCl_4_ in colloidal solutions of Fe oxide NPs in the presence of a number of different chemical substances [27], but this approach is also not free of residual contamination problems and requires sophisticated and costly equipment.

Pulsed laser ablation is an alternative nanofabrication pathway capable of producing nanostructures with various geometries, including the core-shell one. This approach is based on a natural production of nanoclusters during the action of laser radiation on a solid target [28]. When produced in a gaseous medium, the nanoclusters can be deposited on a substrate to form a thin nanostructured film [29], while pulsed laser ablation in liquids (PLAL) leads to the release of nanoclusters into the liquid medium to form a colloidal solution [30]. A high efficiency and cleanness of this method made it highly suitable for a plethora of applications in biomedicine [3,31,32]. Ultrashort laser ablation (femtosecond (fs) or picoseconds (ps)) has demonstrated the most efficient control of NPs among PLAL methods (see examples in [33,34,35]). Using these approaches, we recently synthesized a variety of nanomaterials, including Au [8,33], Si [36], TiN [37,38], organic materials [39], and demonstrated their great potential for biomedical tasks.

PLAL has already been explored in the synthesis of Fe-Au NPs with diverse geometries. Barcikowski et al. ablated Au-Fe alloy target and found that structure of the produced core-shell NPs depends on NPs size and nature of the liquid media [40,41], while Amendola et al. demonstrated that nanosecond laser ablation from an Au-Fe alloy target or layers of alternating Au and Fe thin films in water and ethanol results in the formation of Fe-Au NPs of alloy or core-shell geometry [42,43]. It was shown that such NPs have remarkable magnetic and plasmonic properties and are promising for magnetic resonance imaging (MRI), SERS, and computer tomography (CT) applications [42].

Herein, we further extend the capabilities of laser-ablative methodology toward the synthesis of core-satellite magneto-plasmonic structures. Our approach is based on fs laser ablation from Fe target in aqueous colloidal solutions of small (<10 nm) Au NPs, preliminarily prepared by PLAL. We show that the formed Fe*_x_*O*_y_*@Au (*x*, *y*—variables) core-satellites exhibit good magnetic properties and significant optical extinction in the window of biological tissue transparency. We then use different polymers to coat the core-shell nanocomposites and show that such a coating leads to their colloidal stabilization even under storage in high ionic strength solutions. Such nanocomposites are promising for multimodal magneto-optical biomedical applications.

## 2. Materials and Methods

### 2.1. Synthesis of Nanoparticles

**Figure 1:**
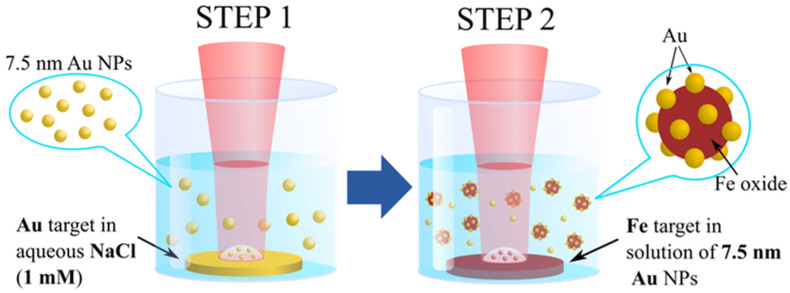
Schematic diagram of two-step PLAL synthesis of core-satellite Au-Fe NPs.

For the synthesis of Fe*_x_*O*_y_*@Au core-satellite nanocomposites we modified a two-step method of fs PLAL, which was previously developed by our group for the preparation of Gold-Silicon core-shell NPs [44,45]. A schematic diagram of the synthesis procedure is shown in Figure 1. During the first step we synthesized Au NPs with mean size 7.5 nm using radiation from the Yb:KGW laser (420 fs, 1025 nm, 10 kHz, 50 µJ) focused by a 75 mm lens on surface of a gold target (99.99%, GoodFellow, Delson, QC, Canada) immersed in 10 mL of 1 mM aqueous NaCl solution, and placed 10 mm below the liquid surface. As previously demonstrated [46,47], the addition of NaCl to water improves colloidal stability of laser-synthesized Au NPs and provides in situ control of the mean size of Au NPs by adjustment of NaCl concentration. The Au target was continuously moved perpendicular to the laser beam at a speed of 2 mm/s to avoid ablation from the same point. Duration of the synthesis was 20 min. During the second step of the synthesis, a metallic Fe target (99.99%, GoodFellow, Delson, QC, Canada) was placed at the bottom of the glass vessel filled with the colloidal solutions of Au NPs prepared in the first step and laser ablation was performed with the same laser, but with slightly modified parameters (repetition rate 5 kHz, pulse energy 100 μJ) for 15 minutes. Ablated mass concentrations of Au and Fe were 0.1 and 0.05 g/L, respectively.

Non-reacted Au NPs were eliminated by magnetic separation step. For this a NdFeB magnet was placed for 5 min next to the bottom of the test tube filled with the colloidal solutions of NPs. The magnet attracted magnetic fraction of NPs to the bottom, while nonmagnetic NPs remained dispersed in the solution. Then the solution from the upper part of the test tube was discarded leaving about 20 μL at the bottom and the test tube was refilled with fresh 1 mM aqueous NaCl solution. The NPs remaining in the test tube were re-dispersed by an ultrasonication step. The procedure was repeated two times to ensure good separation of NPs. The obtained solutions were used for further experiments and characterization. 

### 2.2. Coating of Nanoparticles

Polyethylenimine (PEI) branched 25 kDa, polyallylamine (PAA) hydrochloride 17.5 kDa, carboxymethyl-dextran (CMD) sodium salt, polyacrylic acid (PAAC) sodium salt 5.1 kDa, polyethylene glycol-silane (PEG-silane) 5kDa, ammonium hydroxide 30% were purchased from Sigma-Aldrich (St. Louis, MO, USA). 

NPs coating by CMD, PAAC, PAA, and PEI polymers was performed by coincubation procedure. Total of 200 µL of NPs suspension was mixed with 200 µL of 10% *w*/*v* solution of a polymer in distilled water. Then, the mixture was ultrasonicated, heated to 80 °C for 1 h, cooled to room temperature and the NPs were washed three times with water by centrifugation (20,000 g, 20 min). 

NPs coating by PEG was performed by PEG-silane hydrolysis. For this, 1 mg of NPs was centrifuged at 20,000 g for 20 min and redispersed in dry ethanol. After that 10 µL of 30% hydroxide ammonia and 50 µL of water were added to the suspension and NPs were thoroughly ultrasonicated. Then 100 µg of PEG-silane in 100 µL of dry ethanol was added dropwise, the tube was shaken and heated to 60 °C for 2 h. Then, NPs were washed by centrifugation (20,000 g, 20 min) forming unreacted chemicals 1 time with ethanol and 2 times with water.

### 2.3. Characterization of Nanoparticles

Morphology, structure, size, and composition of the synthesized NPs were characterized by a high resolution transmission electron microscope (HR-TEM, JEM 3010, JEOL, Tokyo, Japan) operating at 300 kV and by a scanning electron microscope (SEM, MAIA 3, Tescan, Brno, Czech Republic) operating at 0.1–30 kV coupled with an energy dispersive spectroscopy (EDS) detector (X-act, Oxford Instruments, Abingdon, UK). Samples for the electron microscopy were prepared by dropping 1 μL of a NPs solution onto a carbon-coated copper grid (for HR-TEM imaging) or onto a cleaned silicon substrate (for SEM imaging) and subsequent drying at ambient conditions.

ζ–potential and hydrodynamic size were measured using a Zetasizer ZS instrument (Malvern Instruments, Malvern, UK) capable of measuring dynamic light scattering (DLS). Concentrations of NPs solutions were determined by measuring the ablation target weight before and after the ablation step and by dividing this mass difference by the ablation liquid volume.

Optical extinction spectra were measured by a UV–VIS spectrophotometer (UV–2600, Shimadzu, Kyoto, Japan) using plastic cuvettes with 10 mm optical path length (for bare NPs) or in 100 µL volume of 96-well plastic plate using Infinite M1000Pro (Tecan, Männedorf, Switzerland) microplate reader (for polymer coated NPs).

Magnetic properties of the NPs were measured using a superconducting quantum interference device (MPMS XL SQUID, Quantum Design, San Diego, CA, USA) magnetometer. Magnetic curves M(H), where M is the magnetization and H is the applied magnetic field, were measured at T = 300 and 5 K. The zero-field cooling/field cooling (ZFC/FC) measurements at H = 100 Oe were also performed on the SQUID. In this purpose, the ZFC temperature dependences of the magnetization were measured by cooling the sample from 325 to 5 K and then applying a field of 100 Oe to record the magnetization, while the sample was heated from 5 to 325 K. The field cooled (FC) measurements were made with a field of 100 Oe applied during the cooling process. 

### 2.4. Calculation of Optical Properties

The extinction spectra of NPs were simulated using the electromagnetic Mie theory, in which the numerical solutions are presented as infinite series of several combinations of Bessel functions [48,49]. The numerical model was based on the implementation of Yang’s algorithm [49] for multilayered spheres [50,51] with the possibility of calculating the optical response of an ensemble of independent particles with a normal size distribution. Thus, the analytical solution for the light scattering by a multilayered sphere was calculated by expressing the electromagnetic field inside each layer of the sphere as a linear combination of the inward and outward travelling waves. Each layer was characterized by a size parameter *x_i_ =* 2*πn_m_r_i_*/*λ = kr_i_* and by a relative refractive index *m_i_ = n_i_*/*n_m_*, *i =* 1, 2, …, *L*. Here, *λ* is the incident wavelength in vacuum, *r_i_* is the outer radius of the *i*th layer, *k* is the propagation constant, and *n_m_* and *n_i_* are the refractive index of the medium outside the particle and its *i*th component, respectively, and *L* is the total number of layers. *L* = 1 for spherical Au and Fe oxide NPs, while *L* = 2 for core-satellites.

## 3. Results and Discussion

### 3.1. Synthesis and Structural, Optical and Magnetic Characterizations of Nanomaterials

Core-satellites were synthesized by a two-step fs PLAL as described in Experimental Section and schematically shown in Figure 1. The prepared colloids of nanocomposites were almost black with a reddish-violet tint. To remove potentially unreacted Au NPs from the colloid we separated magnetically active NPs by applying external magnetic field. Before TEM and EDS imaging the colloidal solutions were additionally kept in a dialysis tube (Serva Visking^®^ 44120, pore diameter ca. 2.5 nm, buffer volume 3 L, Heidelberg, Germany) for 20 h to remove small amorphous iron oxide flakes.

**Figure 2:**
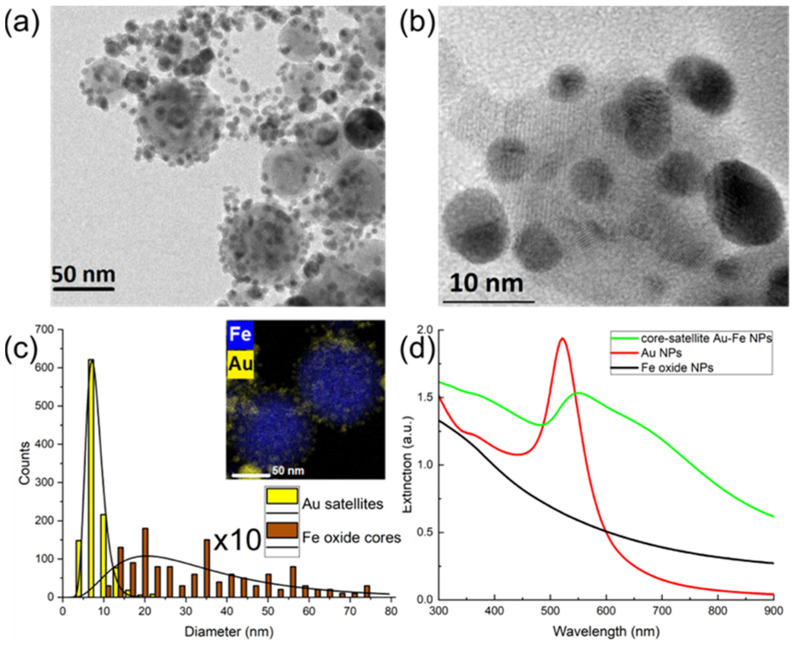
Characterization of core-satellite Au-Fe NPs. (**a**) TEM image of the NPs. (**b**) HR-TEM image of the NPs. (**c**) Size distribution of Au satellites (yellow) and Fe oxide cores (brown) (note that amount of cores is multiplied by 10). The inset demonstrates elemental mapping (Fe—blue, Au—yellow) of the NPs measured by EDX technique. (**d**) Optical extinction spectra of Fe oxide NPs (black curve), Au NPs (red curve) and core-satellite Au-Fe NPs (green curve).

The analysis of TEM images (Figure 2a) showed that most NPs had core-satellite architectures with higher contrast of satellites, which evidences the presence of Fe and Au in the core and satellites, respectively. At the same time, HR-TEM studies clearly demonstrated polycrystalline structure of both the core and the satellites (Figure 2b). Figure 2c (inset) presents the chemical elemental mapping obtained by EDS technique in HR-TEM mode. The figure confirms that, the core was formed uniquely by iron-based compounds, while Au was presented only in satellites. As follows from size histogram depicted in Figure 2c, the size of Fe-based cores varied between 10 and several tens of nm with the mean size 30 nm, while the mean size of Au satellites was 7.5 nm. It should be noted that the core-shells nanostructures with Au core and Fe-based shell were also formed (data not shown), when large (>10 nm) Au NPs were present in the colloid during the ablation of Fe target. Similar tendency was earlier observed and explained for the synthesis of Fe oxide@Au core@shell nanostructures by laser ablation of Au-Fe alloy targets in water [52].

Optical examination of the colloidal solutions (Figure 2d) revealed that the core-satellites exhibited a strong extinction peak associated with the excitation of plasmon resonance in Au NPs [4]. The position of this peak (green line in Figure 2d) was red shifted compared to the plasmonic feature of pure Au NPs centered at 521 nm (red line in Figure 2d). Indeed, the plasmonic peak of the core-satellites was centered at 550 nm and had a broad shoulder extending over 800 nm, which largely overlaps with the optical window of relative tissue transparency (650–950 nm). This broadening of the plasmonic feature can have several origins, including the hybridization and coupling of plasmonic modes between Au NPs in their aggregates [53], the elongation of Au NPs [54], and the formation of the composite core-satellite nanostructures. The coupling between plasmonic modes of individual Au NPs could partly contribute to the spectrum broadening, although it cannot fully explain it, because for Au NPs the plasmonic coupling can redshift the position of the plasmon resonance up to 100 nm (to 620–630 nm) [55], while we observed broadening of the plasmonic feature up to 800 nm. The elongation of Au NPs can be ruled out because TEM images (Figure S1) demonstrate that Au NPs are almost perfectly spherical, and their optical spectrum (Figure 2d, red line) has sharp plasmonic peak at 520 nm without any broadening. Therefore, we can conclude that the origin of the observed broadening of the plasmonic feature (Figure 2d, green line) must include the formation of core-satellites nanostructures, in which the core and the satellites are formed by dielectric Fe oxide and Au, respectively.

We then carried out a series of calculations to simulate the observed plasmonic response. The numerical model was based on the implementation of Yang’s algorithm for multilayered spheres. In the case of simple one-component NPs (pure Au or Fe oxide NPs) the solution corresponds to the classical Mie theory. In the case of core-satellites, we adopted the effective-medium Maxwell-Garnett approximation describing satellites as a layer (shell) with effective optical properties [56,57]. The effective shell consists of a matrix medium (water) with Au NPs as inclusions. The resulted spectra along with the calculation parameters are shown in Figure S2. The simulated extinction spectra are in a reasonable agreement with the experimental results. Good agreement was observed for the curves calculated for bare Fe oxide NPs and Au NPs. For the core-satellites, even though the applied approximation disregards coupling between resonant plasmonic modes of Au satellites, the general trend is still rather well reproduced, particularly the peak and the slow decay of the extinction coefficient with increase of wavelength.

**Figure 3:**
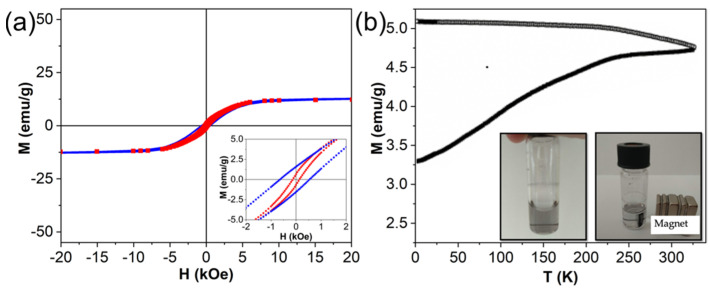
Magnetic characterization of core-satellite Au-Fe NPs. (**a**) Magnetic hysteresis loops measured at 5 K (blue) and 300 K (red). The inset is an expanded view of the low-field region; (**b**) ZFC-FC curves. The inset represents colloidal solutions of the NPs without magnetic field (**left**) and attracted by the magnet (**right**).

We also investigated magnetic properties of the synthesized NPs. The results for pure laser-synthesized Fe oxide NPs and for the core-satellite nanostructures are summarized in Table 1. Hysteresis loops recorded at 5 K and 300 K are presented in Figure 3a. The hysteresis loops at both temperatures were open and clearly showed ferromagnetic behavior of the NPs. The coercive field at 5 K was slightly larger than at 300 K. The saturation magnetization (M_S_) of core-satellites (M_S_ = 12.3 emu/g at 300 K) was significantly lower than M_S_ of bulk magnetite (92 emu/g) [58] or bulk maghemite (76 emu/g) [59]. The M_S_ of pure laser-ablated Fe oxide NPs was 44.7 emu/g at 300 K. The decrease of the saturation magnetization values in comparison to bulk materials is typical for magnetic NPs [60] and it depends on many factors, such as crystal structure, size, shape, and surface properties [61]. The differences in saturation magnetization can also be due to spin disorder at the surface of Fe oxide NPs [62]. Moreover, the decrease of M_S_ of core-satellite NPs was also caused by a relative decrease of magnetic material per gram of the nanocomposites due to presence of Au satellites.

It is worth mentioning that magnetization hysteresis loops recorded at 5 K have a clear negative shift from the origin along the field axis. This observation indicates the presence of an exchange bias field (H_EB_), which originates from the interface exchange coupling between ferro (ferri)/antiferromagnetic [63]. The presence of exchange bias field can also explain the high value of coercivity for the investigated NPs. Different phases of Fe oxide in the nanocomposites, such as magnetite or maghemite, which are ferrimagnets, and wustite (FeO), which is antiferromagnet, could be responsible for the exchange bias fields [64]. ZFC/FC curves of the core-satellite nanostructures are presented in Figure 3b. No well-defined maximum in the ZFC curve was found. This fact indicates that the NPs had blocking temperatures above 325 K and exhibit ferromagnetic behavior at room temperature.

Structural and colloidal stability of the synthesized nanocomposites are very important for the projected biomedical applications. To check the stability and find out whether bonds between Fe oxide cores and Au satellites can withstand high ionic strength conditions of physiologically relevant liquids, we incubated the laser-synthesized nanocomposites in aqueous solutions of 1 M NaCl and in phosphate buffered saline (PBS) at pH 7.4. Colloidal stability was poor in both liquids: hydrodynamic size of the nanocomposites incubated at 37 °C doubled after just 5 min (detailed kinetic of aggregation over the course of 30 min is shown in Table S1). This result is not surprising since the colloidal stabilization mechanism of bare laser-synthesized NPs is based on electrostatic repulsion, while surface charges are effectively screened in high ionic strength liquids. However, it is very important that the aggregation of the nanostructures was reversible since it was possible to restore optical extinction spectra of the nanocomposites by simple ultrasonication step. The reproducibility of the optical extinction spectra after re-dispersion by ultrasonication means that the nanostructures preserved their structural stability in high ionic strength solvents, although they were colloidally unstable.

### 3.2. Polymer Coating

To improve colloidal stability in high ionic strength liquids and to additionally ensure structural stability of the nanocomposites we covered them using several different biocompatible polymer coatings, namely: PEG-silane, PAAC, CMD, PEI, and PAA.

**Figure 4:**
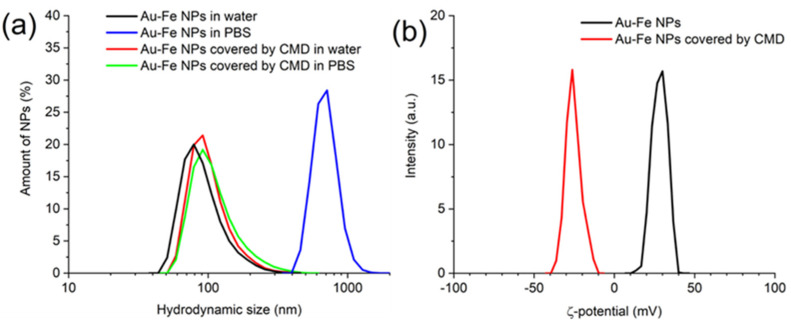
(**a**) DLS size histograms of the core-satellite Au-Fe NPs after incubation for 24 h in water and PBS. Black curve—bare NPs incubated in water, blue curve—bare NPs incubated in PBS, red curve—CMD covered NPs incubated in water, green curve—CMD covered NPs incubated in PBS. (**b**) ζ-potential of bare (black) and CMD covered (red) core-satellite Au-Fe NPs.

Table S2 summarizes NPs properties after the coating procedure. Efficiency of coating was confirmed by the increase of colloidal stability in PBS buffer and change of ζ-potential of NPs. The best colloidal stability was achieved using negatively charged polymer CMD, while other coatings provided less colloidal stability, or the nanocomposites even aggregated during the coverage procedure (Table S2). The CMD-coated nanocomposites only slightly change their hydrodynamic size after incubation for 24 hours in PBS from 105 ± 39 nm to 116 ± 52 nm (Figure 4a, Table S2). The coverage by negatively charged CMD polymer changed ζ-potential of the NPs from +28 ± 5 mV to −25 ± 4.8 mV (Figure 4b) and slightly increased their hydrodynamic size (Figure 4a), which additionally confirms the efficiency of the coating procedure.

### 3.3. Formation Mechanism

Our experiments showed that ζ-potential was negative for bare laser-synthesized Au NPs (−27 mV) and positive for both bare Fe oxide NPs (+30 mV) and core-satellite Au-Fe NPs (+28 mV). Therefore, one can suppose that electrostatic interaction can potentially be a major formation mechanism of the nanocomposites. To test this possibility, we separately synthesized bare Au and Fe oxide NPs by a similar laser-ablative procedure (in this case the Fe target was ablated in deionized water in the absence of Au colloids). Then the Au and Fe-based NPs were mixed in a vial followed by vortexing for several minutes and magnetic separation as described in Materials and Methods section. Finally, we compared the mixture of Au and Fe oxide NPs with nanocomposites obtained by PLAL. We observed the presence of core-satellite Fe-Au nanostructures in the mixture (Figure S3). However, there was a substantial difference in optical spectra of the samples prepared by PLAL and by NPs mixing as it is shown in Figure S4. The intensity of plasmonic feature of core-satellites NPs normalized to Fe content was five-folds higher for laser-ablated NPs compared to that of NPs prepared by mixing. This highly intense plasmonic signal from laser-synthesized Fe-Au NPs originated from Au NPs attached to Fe oxide cores, because all free Au NPs were removed from colloids by magnetic separation step, while possible differences in concentration of Fe content were accounted by spectra normalization. Therefore, the efficiency of the nanocomposite formation was significantly higher for the laser-ablative synthesis than for mixing of oppositely charged Au and Fe oxide NPs. This fact suggests that there are other mechanisms leading to the formation of nanocomposites during ablation of Fe target in Au NPs colloids rather than simple electrostatic interaction of oppositely charged NPs.

In our opinion, the formation of the core-satellites starts from an attraction of small Au NPs to the forming Fe oxide NPs. The attraction between NPs is originated from several mechanisms including an electrostatic interaction between oppositely charged Au and Fe oxide NPs, Van der Waals forces, and hydrophobicity of both Au and Fe oxide NPs. For the formation of stable core-satellites, Au NPs should arrive at the surface of Fe oxide NPs, while the latter are relatively hot. As a result, these small Au NPs fill the potential well at the interface with water and settle down at the surface of Fe oxide NPs. In this way, core-satellites are formed during a limited cooling time of Fe oxide NPs. This explanation is supported by several observations. First, a simple mixing of Au and Fe oxide NPs is less effective for the formation of core-satellite structures than the laser-ablative synthesis. Second, the satellites in the nanocomposites are predominantly formed by the smallest Au NPs presented in initial gold colloid. Third, larger Fe oxide cores tend to form more core-satellite structures than smaller cores (Figure 2a,b). The second observation can be rationalized by higher speed of NPs Brownian motion of smaller NPs (the speed depends on the size as *r*^−3^). Therefore, small Au NPs move much faster than the large ones and have higher probability of reaching the surface of the Fe oxide NPs during the cooling time. The last observation can be explained by longer cooling time of larger Fe oxide NPs, which gives Au satellites more time to reach the Fe oxide cores. Moreover, note that in our laser ablative setup some of already formed nanostructures are re-irradiated by the laser beam. This re-irradiation can also contribute to the formation of core-satellites in a sintering-like process [65].

Thus, we managed to synthesize stable Au-Fe core-satellite nanocomposites, which combine prominent magnetic response with the generation of plasmonic absorption peak within optical transparency window. At the same time, unique laser-ablative synthesis guarantees purity of the nanocomposites and free access to both Au and Fe surfaces for further biofunctionalization. There is yet another advantage of the Fe*_x_*O*_y_*@Au core-satellite nanocomposites, which potentially can solve the problem of residual accumulation of the nanomaterials in an organism. Fe oxide cores can slowly degrade in physiological conditions and be excreted from an organism, while Au satellites are small enough to be eliminated through renal glomerular filtration mechanism [66]. Therefore, both components of the core-satellites can be naturally removed from an organism, which promises a great advancement in the field of nanomedicine, although this clearance pathway is still to be verified in further research. Combination of these properties makes laser-synthesized Fe*_x_*O*_y_*@Au nanocomposites highly suitable for both established and novel theranostic magneto-plasmonic modalities for cancer therapy.

## 4. Conclusions

In conclusion, we synthesized Fe*_x_*O*_y_*@Au core-satellite nanocomposites by using a purely physical method of laser ablation in liquids, which insures a high purity of the obtained nanomaterials and the absence of any residual contamination. We experimentally determined optical, structural and magnetic properties of the nanocomposites and elaborated a numerical model, which describes their optical properties. We showed that such nanoformulations combine a strong magnetic response and a good optical extinction within optical transparency window. Additionally, we demonstrated that the surface coating of laser-synthesized nanocomposites with biopolymer CMD can significantly improve its colloidal stability in biologically relevant fluids that is very important for the projected biomedical applications, which are now in progress. From a broad perspective, our research provides a novel extremely pure magneto-plasmonic platform for biomedical nanotheranostics.

## Figures and Tables

**Table 1:** Magnetization values of Fe oxide NPs and core-satellite Au-Fe NPs at 5 K and 300 K. M_S_—magnetization saturation, H_(left)_—intersections on the field axis at increasing and decreasing fields (H_(right)_), H_C_—coercive field, H_EB_—exchange bias field.

Type of NPs	T, K	H_(left)_, Oe	H_(right)_, Oe	H_C_, Oe	H_EB_, Oe	M_S_, emu g^−1^
Fe oxide	5	−1572	624	1098	−474	50.5
	300	−109	110	109.5	~0	44.7
Core-satellite	5	−643	534	588.5	−54.5	12.9
	300	−134	132	133	0	12.3

## Data Availability

Data is available on reasonable request from the corresponding author.

## Supplementary Materials

The following are available online at https://www.mdpi.com/article/10.3390/nano12040649/s1. Figure S1: Typical TEM image of laser-synthesized Au NPs. Figure S2: Calculated extinction spectra of bare Au NPs (red line), Fe oxide NPs (black line), and core-satellite Au-Fe NPs (green line). The following parameters were used for calculations: Au NPs diameter 8 nm; Fe oxide NPs diameter 60 nm, optical constants: *n* = 2, k = 0.129; Core-satellite NPs core diameter 60 nm, effective shell thickness 10 nm, optical constants *n* = 2.4, k = 2.9. Figure S3: STEM image of nanostructures, obtained by mixing of negatively charged laser-ablated Au NPs and positively charged laser-ablated Fe oxide NPs. Figure S4: Optical extinction spectra of core-satellite nanostructures obtained by mixing of Au NPs and Fe oxide NP (black) and by laser ablation of iron target in colloidal solution of Au NPs (red). Extinction spectrum of laser-ablated Fe oxide NPs was subtracted from both spectra. Table S1: DLS size of the bare core-satellite Au-Fe nanocomposites, incubated in PBS (pH 7.4) at 37 °C. Table S2: DLS size (number distribution) and ζ-potential of the core-satellite Au-Fe NPs, coated with different polymers after incubation for 24 h in water and PBS.
