# Supplementary material for: Laser-Ablative Synthesis of Ultrapure Magneto-Plasmonic Core-Satellite Nanocomposites for Biomedical Applications
Source: Nanomaterials (Basel). 2022 Feb 15;12(4):649. doi: 10.3390/nano12040649 (PMC8880494; doi:10.3390/nano12040649)
Supplement: Supplementary file 1 [file nanomaterials-12-00649-s001.zip › nanomaterials-1562049-supplementary.pdf]

Supporting Information

Laser-Ablative Synthesis of Ultrapure Magneto-Plasmonic Core-Satellite Nanocomposites for Biomedical Applications

Anton A. Popov [1,2,*], Zaneta Swiatkowska-Warkocka [3], Marta Marszalek [3], Gleb Tselikov [2,4], Ivan V. Zelepukin [1,5], Ahmed Al-Kattan [2], Sergey M. Deyev [1,5], Sergey M. Klimentov [1], Tatiana E. Itina [6] and Andrei V. Kabashin [1,2,*]

[1] Institute of Engineering Physics for Biomedicine (Phys-Bio), Moscow Engineering Physics Institute, 115409 Moscow, Russia; ivan.zelepukin@gmail.com (I.V.Z.); deyev@ibch.ru (S.M.D.); smklimentov@mephi.ru (S.M.K.)

[2] Laboratory of Lasers Plasmas and Photonic Processing, CNRS, Aix-Marseille University (Campus of Luminy), 13288 Marseille, France; celikov@physics.msu.ru (G.T.); ahmed.al-kattan@univ-amu.fr (A.A.-K.)

[3] Institute of Nuclear Physics, Polish Academy of Sciences, 31342 Kraków, Poland; zaneta.swiatkowska@ifj.edu.pl (Z.S.-W.); marta.marszalek@ifj.edu.pl (M.M.)

[4] Center for Photonics and 2D Materials, Moscow Institute of Physics and Technology, 141700 Dolgoprudny, Russia

[5] Shemyakin–Ovchinnikov Institute of Bioorganic Chemistry, Russian Academy of Sciences, 117997 Moscow, Russia

[6] Hubert Curien laboratory (UMR CNRS 5516), Jean Monnet University, 42000 Saint-Etienne, France; tatiana.itina@univ-st-etienne.fr

* Correspondence: aapopov1@mephi.ru (A.A.P.); andrei.kabashin@yahoo.com (A.V.K.)

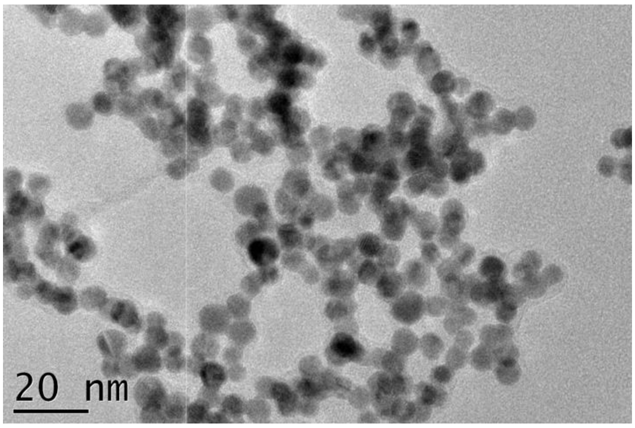

Figure S1. Typical TEM image of laser-synthesized Au NPs.

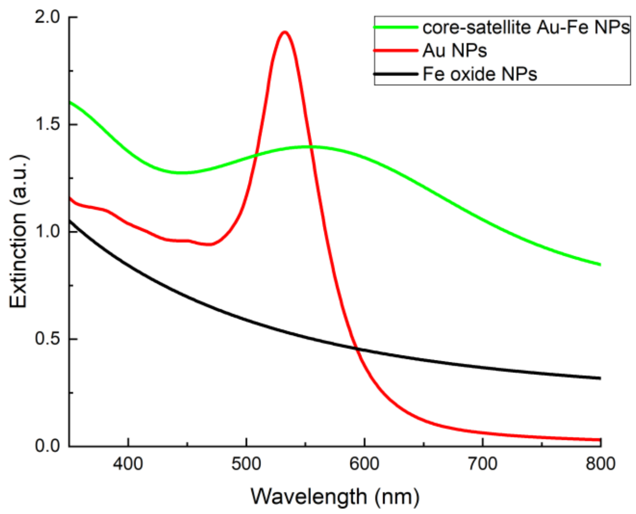

Figure S2. Calculated extinction spectra of bare Au NPs (red line), Fe oxide NPs (black line) and core-satellite Au-Fe NPs (green line).

The following parameters were used for calculations:
Au NPs diameter 8 nm;
Fe oxide NPs diameter 60 nm, optical constants: n = 2, k = 0.129;
Core-satellite NPs core diameter 60 nm, effective shell thickness 10 nm, optical constants n = 2.4, k = 2.9.

Table S1. DLS size of the bare core-satellite Au-Fe nanocomposites, incubated in PBS (pH 7.4) at 37°C.

Time of incubation, min	Mean size, nm	Standard deviation, nm
0	205	108
1	275	121
3	332	143
5	450	277
15	830	212
30	961	247

Table S2. DLS size (number distribution) and ζ-potential of the core-satellite Au-Fe NPs, coated with different polymers after incubation for 24 hours in water and PBS.

Coating	ζ-potential, mV	Hydrodynamic size in water, nm	Hydrodynamic size in PBS, nm
Uncoated	+28 ± 5	95 ± 38	701 ± 144
Carboxymethyldextran	−25 ± 4.8	105 ± 39	116 ± 52
Polyacrylic acid	−46.7 ± 5.4	100 ± 34	131 ± 56
Polyallylamine	+20 ± 4.8	209 ± 70	1192 ± 297
Silane-PEG	−9.8 ± 5.4	167 ± 39	273 ± 133
Polyethyleneimine	+34.9 ± 4.7	163 ± 45	766 ± 190

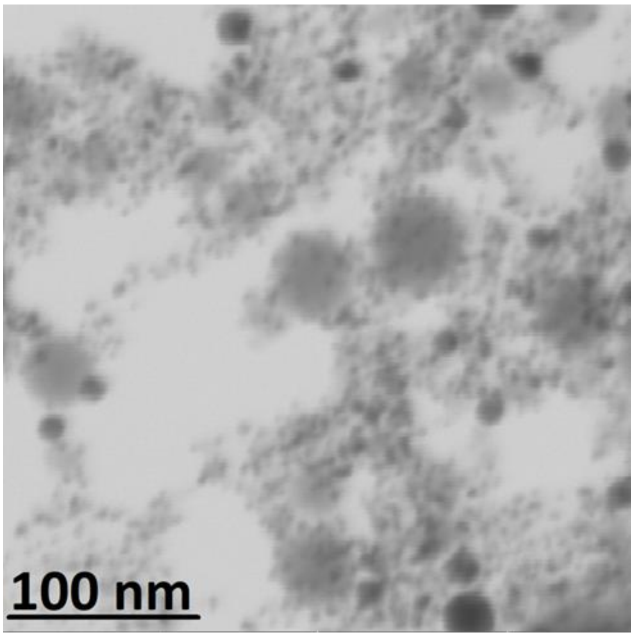

Figure S3. STEM image of nanostructures, obtained by mixing of negatively charged laser-ablated Au NPs and positively charged laser-ablated Fe oxide NPs.

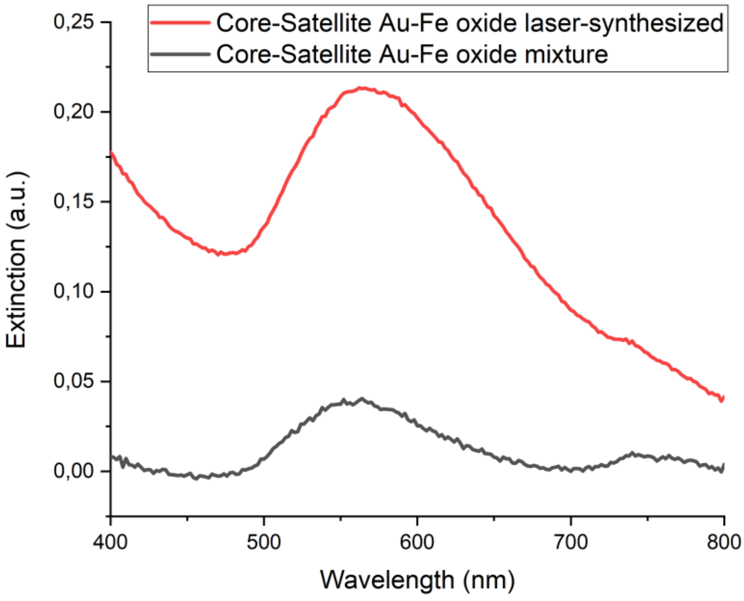

Figure S4. Optical extinction spectra of core-satellite nanostructures obtained by mixing of Au NPs and Fe oxide NP (black) and by laser ablation of iron target in colloidal solution of Au NPs (red). Extinction spectrum of laser-ablated Fe oxide NPs was subtracted from both spectra.
